# The Tri-Steps Model of Critical Conditions in Intensive Care: Introducing a New Paradigm for Chronic Critical Illness

**DOI:** 10.3390/jcm13133683

**Published:** 2024-06-24

**Authors:** Valery V. Likhvantsev, Levan B. Berikashvili, Mikhail Ya. Yadgarov, Alexey A. Yakovlev, Artem N. Kuzovlev

**Affiliations:** Federal Research and Clinical Center of Intensive Care Medicine and Rehabilitology, Moscow 107031, Russia; levan.berikashvili@mail.ru (L.B.B.); mikhail.yadgarov@mail.ru (M.Y.Y.); ayakovlev@fnkcrr.ru (A.A.Y.); artem_kuzovlev@fnkcrr.ru (A.N.K.)

**Keywords:** chronic critical illness, persistent inflammation, immunosuppression, and catabolism syndrome (PICS), eICU-CRD

## Abstract

**Background:** The prevailing model for understanding chronic critical illness is a biphasic model, suggesting phases of acute and chronic critical conditions. A major challenge within this model is the difficulty in determining the timing of the process chronicity. It is likely that the triad of symptoms (inflammation, catabolism, and immunosuppression [ICIS]) could be associated with this particular point. We aimed to explore the impact of the symptom triad (inflammation, catabolism, immunosuppression) on the outcomes of patients hospitalized in intensive care units (ICUs). **Methods:** The eICU-CRD database with 200,859 ICU admissions was analyzed. Adult patients with the ICIS triad, identified by elevated CRP (>20 mg/L), reduced albumin (<30 g/L), and low lymphocyte counts (<0.8 × 10^9^/L), were included. The cumulative risk of developing ICIS was assessed using the Nelson–Aalen estimator. **Results:** This retrospective cohort study included 894 patients (485 males, 54%), with 60 (6.7%) developing ICIS. The cumulative risk of ICIS by day 21 was 22.5%, with incidence peaks on days 2–3 and 10–12 after ICU admission. Patients with the ICIS triad had a 2.5-fold higher mortality risk (*p* = 0.009) and double the likelihood of using vasopressors (*p* = 0.008). The triad onset day did not significantly affect mortality (*p* = 0.104). Patients with ICIS also experienced extended hospital (*p* = 0.041) and ICU stays (*p* < 0.001). **Conclusions:** The symptom triad (inflammation, catabolism, immunosuppression) during hospitalization increases mortality risk by 2.5 times (*p* = 0.009) and reflects the chronicity of the critical condition. Identifying two incidence peaks allows the proposal of a new Tri-steps model of chronic critical illness with acute, extended, and chronic phases.

## 1. Introduction

The concept of chronic critical illness (CCI), first conceptualized nearly 40 years ago by Girard and Raffin [1], is not currently recognized in international medical disease classifiers but has increasingly captured the interest of the medical community. Over the years since CCIs were characterized, critical care medicine has advanced significantly, enabling the survival of patients who previously could not have survived even a day under intensive care [2]. This progress has come at a cost, notably the rise in patients suffering from this new condition known as chronic critical illness [3,4].

The primary challenge at this stage in the evolution of medical science is the lack of a definitive classification for this nosological entity [5]. It is particularly difficult to pinpoint the exact moment when the acute phase of a critical condition transitions into a chronic phase [6]. Moreover, the characteristics of the acute phase cannot be overlooked, as the etiological factor therein is dominant in determining the severity of the patient’s condition, the prognosis of the disease, and its duration. This two-phase model of CCI brings us to a global issue in defining CCI: stratification based on etiological features. Etiological stratification implies that patients with different etiologies of acute conditions have fundamentally different timelines from the onset of illness for diagnosing CCI [7]. This creates a precedent for a variable point of chronicity of the acute process. With this approach, the only commonalities among CCI patients are high mortality [8,9] and prolonged stays in intensive care units (ICUs), leading to a variety of definitions [10,11,12,13,14,15,16,17,18,19]. Clearly, the absence of a universally accepted definition makes it impossible to conduct any epidemiological research related to CCI, which in turn hinders the development of medical strategies for the prevention and treatment of CCI.

A particularly interesting perspective on CCI involves examining its pathophysiological aspects [20,21]. Special attention has been given to the persistent inflammation, immunosuppression, and catabolism syndrome (PIICS), a concept first introduced in 2012 as a primary cause of late multiorgan failure in surgical ICU patients [22]. Unfortunately, the definition of PIICS also suffers from a lack of standardization in the markers of each of the three symptoms [4,22,23,24,25]. Nonetheless, it is important to note that PIICS encompasses not only the presence of inflammation, immunosuppression, and catabolism but also a time interval during which the patient’s condition requires ICU hospitalization [22,26]. In any definition, this period is not less than 10 days [26]. This approach also appears somewhat unusual because it includes a set of three laboratory markers and a time interval. In the context of CCI stratification and the variable timing of chronicity, the inclusion of additional fixed or variable time points within PIICS poses significant challenges for clinical practice and medical research. Furthermore, the fact that the prolonged use of intensive care methods leads to a systemic inflammatory response [27,28,29,30,31,32] further complicates the issue of timing within PIICS.

The aim of our study was to specifically determine the impact of the triad of symptoms (inflammation, catabolism, and immunosuppression [ICIS]) on the outcomes of patients hospitalized in ICUs.

## 2. Materials and Methods

### 2.1. Data Sources

The primary data source utilized was the eICU Collaborative Research Database (eICU-CRD), developed by Philips Healthcare in collaboration with the Massachusetts Institute of Technology’s Laboratory for Computational Physiology. This database comprises clinical data from 200,859 hospitalizations across 335 ICUs in 208 U.S. hospitals during 2014–2015 [33]. All patient data were anonymized, negating the need for approval by a local ethics committee. One of the authors completed training in “Human Research: Data or Specimens Only Research” and “Conflicts of Interest” and was granted access to the eICU-CRD (eICU) database (certificate numbers: 56653575, 56653561; valid until 21 June 2026).

### 2.2. Selection Criteria

This retrospective cohort study included all adult patients in ICUs who could be evaluated for the presence of ICIS from day 2 to day 21 of hospitalization. Daily ICIS presence was determined based on the simultaneous fulfillment of three criteria: C-reactive protein (CRP) levels above 20 mg/L, albumin levels below 30 g/L, and lymphocyte counts under 0.8 × 10^9^/L [34]. The exclusion criteria were as follows: (1) had an ICU stay of less than 24 h, (2) was readmitted to the ICU, and (3) lacked age or sex information.

### 2.3. Data Extraction

The data were extracted using SQLite version 3.45.2 (https://www.sqlite.org/, accessed on 25 April 2024). The parameters analyzed included the following: (1) general patient information—sex, age, body mass index (BMI), and body weight at admission and discharge; (2) additional information—APACHE IV score, duration and use of mechanical ventilation, use of vasopressors, and lactate levels at admission; (3) main reasons for ICU admission; (4) parameters required for ICIS assessment—CRP, albumin levels, and lymphocyte counts; (5) hospitalization outcomes—hospital and ICU mortality, length of stay in the hospital and ICU, and discharge location. For patients with multiple laboratory measurements on the same day, the earliest values were analyzed. For patients with ICIS, the day of ICIS onset was determined; for others, the last day on which an ICIS assessment was possible was evaluated.

### 2.4. Outcomes

The primary endpoint was the risk of developing ICIS among ICU patients. The secondary endpoints included all-cause mortality and the duration of hospitalization. Additional analyses identified risk factors associated with the development of ICIS. The durations of the ICU and hospital stays were defined as the total lengths of stay in the ICU and hospital, respectively.

### 2.5. Statistical Analysis

The distribution of the data was assessed using the Shapiro-Wilk test. Continuous variables were presented as medians (Mes.) and interquartile ranges (IQRs), while categorical variables were expressed as frequencies and percentages (%). The chi-square test and Fisher’s exact test were used for comparing frequencies; the Mann-Whitney U (Wilcoxon rank-sum) test was applied for comparing continuous variables. The confidence intervals (CIs) for proportions were calculated using the Wilson method. Cumulative risk functions for the development of the ICIS were estimated using the Nelson–Aalen estimator. For censored data, survival tables were constructed, and the Kaplan-Meier method was used to plot survival curves. Differences in survival between groups were assessed using the log-rank test. Independent predictors of ICIS and mortality in this patient cohort were identified using multivariable Cox regression analysis, which revealed adjusted hazard ratios (HRs) accounting for confounding bias and 95% confidence intervals (95% CIs). The optimal cutoff point in the ROC analysis (evaluating the AUROC parameter) was chosen based on the Youden index to maximize sensitivity and specificity. We did not impute missing data. The significance level (two-sided) was set at 0.05. All the statistical calculations were performed using IBM SPSS Statistics v. 29.0 and Stata v. 18.0.

## 3. Results

### 3.1. Patient Characteristics

**Figure 1:**
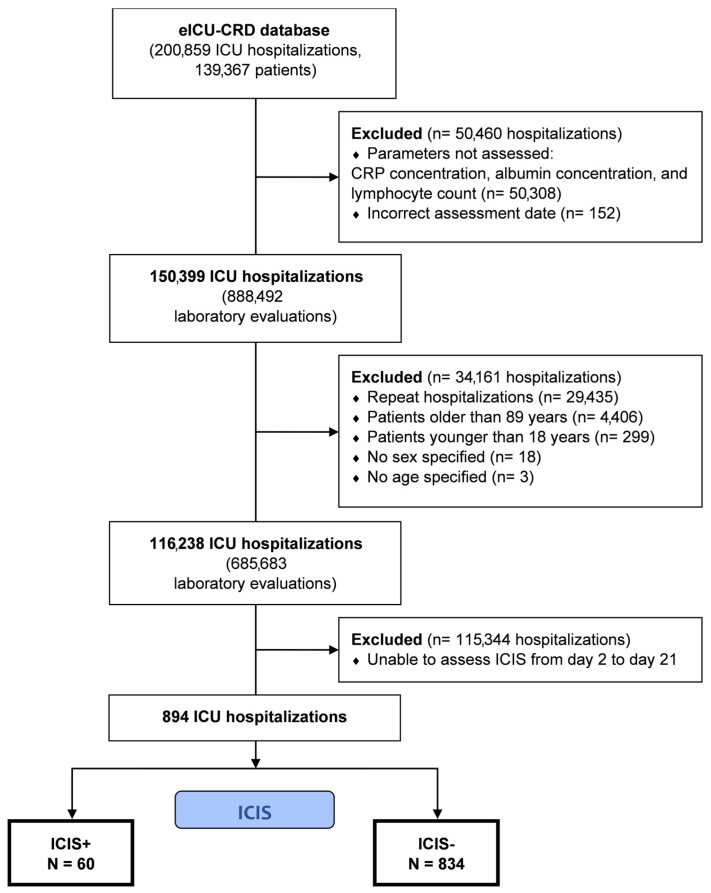
Flowchart of patient selection process employed in this study.

A flowchart of the patient selection process employed in this study is shown in Figure 1. A total of 138,473 patients were sequentially excluded based on the exclusion criteria. The study analyzed 894 patients (485 males, 54%), including 60 patients with ICIS (6.7%). The median age was 63.5 years, and the primary reason for ICU admission was sepsis (30%, Table 1). Mechanical ventilation was administered to 300 patients (34%), and vasoactive drugs were used in 133 patients (15%). The ICU mortality rate was 5.8%, with a median ICU stay duration of 3.6 days (IQR 1.8–8.7). Half of the patients were transferred to another hospital for further treatment or post-discharge rehabilitation (375, 50%).

The strengthening the reporting of observational studies in epidemiology (STROBE) checklist for our paper is provided in Supplementary Material File S1.

### 3.2. ICIS Development Risk

**Figure 2:**
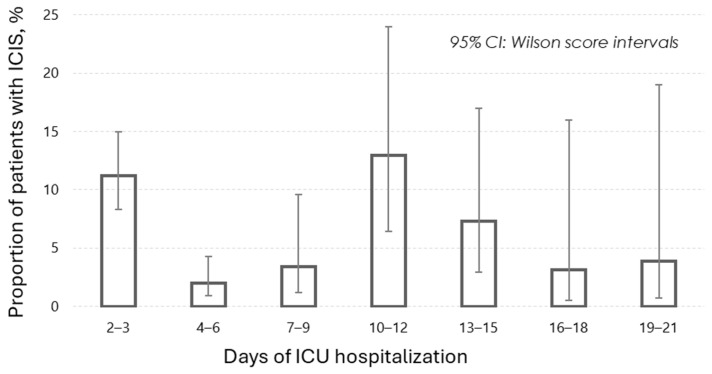
Proportion of patients with ICIS in the period of 2–21 days.

The proportion of patients with ICIS in the study was 6.7%. The median time to ICIS development was 3 days (IQR 2–9). Figure 2 presents a diagram indicating two peaks in the proportion of patients with ICIS: on days 2–3 and days 10–12 of the ICU stay.

**Figure 3:**
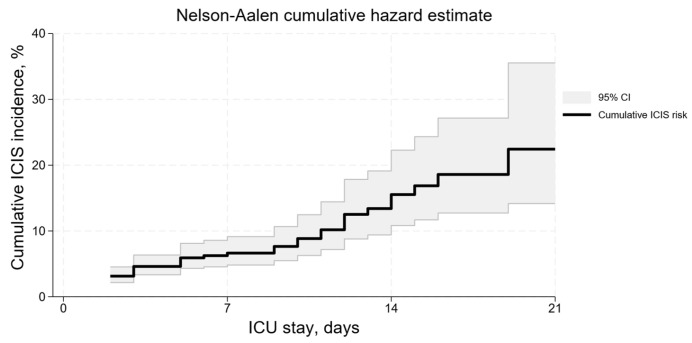
Cumulative risk of ICIS development in a period of 2–21 days.

The cumulative risk of developing ICIS was 6.5% (95% CI 4.8–9.1) by day 7 of the ICU stay and 15.5% (95% CI 10.9–22.3) by day 14. The risk level reached 22.5% (95% CI 14.2–35.6) by day 21 (Figure 3).

### 3.3. ICIS Outcomes

**Figure 4:**
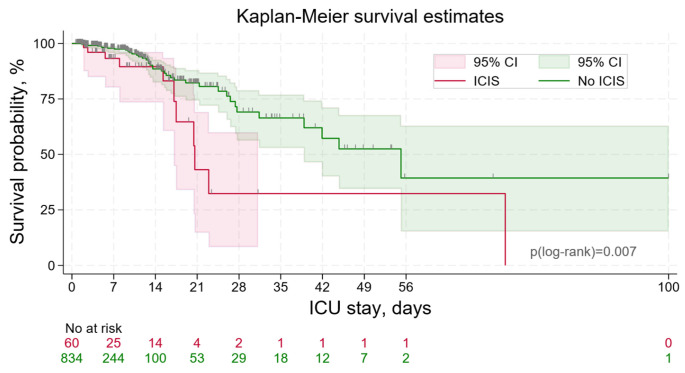
Kaplan-Meier curves showing the impact of ICIS on survival in ICU patients.

Patients with ICIS exhibited markedly higher hospital and ICU mortality rates (18.3% vs. 4.9%, *p* < 0.001) and significantly longer stays in both the hospital (13 days vs. 11 days, *p* = 0.041) and ICU (6.0 days vs. 3.4 days, *p* < 0.001) (Table 1). Kaplan-Meier analysis revealed that patients with ICIS had worse survival in the ICU, with a median survival time of 20.6 days (95% CI 16.3–24.9) compared with 55.2 days (95% CI 36.2–74.1, *p* = 0.007) for patients without ICIS (Figure 4). Cox regression analysis revealed that the development of ICIS was associated with a 2.5-fold increase in the risk of mortality in the ICU (hazard ratio: 2.48 [95% CI 1.25–4.90], *p* = 0.009), and the APACHE IV score was also a risk factor (hazard ratio: 1.012 [95% CI 1.001–1.024], *p* = 0.039). Upon further analysis, it was demonstrated that risk factors for mortality include albumin levels below 30 g/L (*p* = 0.010) and lymphocyte counts below 0.8 × 10^9^/L (*p* = 0.042) but not CRP levels above 20 mg/L (*p* = 0.237). The timing of ICIS onset did not significantly affect mortality risk (hazard ratio: 0.87, [95% CI 0.74–1.03], *p* = 0.104). Furthermore, the absolute risk of requiring vasoactive drugs during hospitalization doubled in patients with ICIS (27% compared to 14%, *p* = 0.008, relative risk 1.9, 95% CI 1.2–3.0).

### 3.4. Risk Factors for ICIS

The baseline characteristics of the patients are presented in Table 1. Patients with ICIS had significantly higher APACHE IV scores upon admission (*p* = 0.017) and were more frequently admitted to the ICU for pancreatitis (*p* = 0.018). According to the multivariable Cox regression analysis, the only independent predictor of developing ICIS was the APACHE IV score (Table 2). An increase in the APACHE IV score by one point was associated with a 1.3% increase in the risk of developing ICIS, and patients with an APACHE IV score of 65 or higher had a 2.36-fold increased risk of developing ICIS (*p* = 0.003).

## 4. Discussion

This study demonstrated that patients with the ICIS triad are 2.5 times more likely to experience a fatal outcome and are twice as likely to require vasoactive drugs than patients without the triad. Notably, mortality is influenced by the presence of the triad itself, not the timing of its onset. The cumulative risk of developing ICIS by day 21 was 22.5%, with two peaks of incidence on days 2–3 and 10–12 of the ICU stay. Patients with ICIS also had longer hospital stays and ICU durations.

Although this study is the first to explore the impact of the early onset of the ICIS triad on patient outcomes, the results align with previously published studies examining chronic critical illness, which emerged on days 10–14 in combination with manifestations of PIICS, as these studies also reported higher mortality in this patient group [9,35,36].

The uniqueness of this work is underscored by several aspects. First, it has been demonstrated for the first time that the presence of the ICIS triad is a risk factor for mortality, regardless of the syndrome’s onset timepoint. This finding challenges the necessity of using the term PIICS and establishing a specific duration of ICU stay as a mandatory component for diagnosing the syndrome associated with adverse outcomes. The emergence of the triad at any point during the ICU stay reflects an unfavorable disease progression. The primary reason for the adverse impact of ICIS on hospitalization outcomes is that this triad reflects disruptions in several physiological processes, such as immune response, nutritional status, and the balance of pro-inflammatory and anti-inflammatory processes. Collectively, these factors can contribute to the development of multiple organ failures and recurrent infectious complications in ICU patients, leading to poorer prognoses for this patient group.

Second, this is the first study to investigate the timing of the onset of the ICIS triad, revealing two distinct peaks on days 2–3 and 10–12 of ICU hospitalization. Interestingly, the second peak coincides with all definitions of PIICS [26] and the most popular definitions of CCI [9,37,38,39,40,41,42,43,44,45]. If we assume that the basis of CCI includes a triad of inflammation, immunosuppression, and catabolism, the peak occurrence of the ICIS triad on days 2–3 suggests that the acute phase becomes chronic by the second day. From a clinical perspective, this assertion seems illogical and contradicts real-world clinical experience. Therefore, there is no established definition of chronic critical illness that would use such short durations of hospitalization [8,9,35,36,37,38,39,40,41,42,43,44,45,46,47,48,49,50,51,52,53,54,55,56,57,58,59,60,61,62,63,64,65,66,67,68,69,70,71,72]. Moreover, it is inaccurate to assert that this triad characterizes an acute process. According to current understanding, individual elements of this triad, such as inflammation, may indicate the severity of an acute condition. However, contemporary perspectives suggest that the simultaneous occurrence of all three pathophysiological processes indicates deeper and more prolonged systemic disturbances than those seen in acute illness [22].

The identified peaks in the diagnosis of the ICIS triad appear to challenge the validity of the two-phase theory of CCI development. Simultaneously, these findings may suggest an alternative principle of critical condition chronicity—a Tri-steps model. Assuming there is an intermediate period between acute and chronic critical conditions, termed as the “extended critical state”, that differs from both acute and chronic states, then the peaks in diagnosing the ICIS triad mark the transition points between these three states—acute, extended, and chronic. In such a model, the severity of the acute critical state is primarily determined by its etiology. The extended critical state represents an intersection of pathophysiological processes triggered by the initial damaging factor and the typical pathological processes that develop due to the prolonged duration of the critical condition and the application of intensive care therapies. The chronic critical state is characterized by the dominance of typical pathological processes that no longer have a direct causal link to the etiology of the initial acute disruption. Within this Tri-steps model, the development of the ICIS triad probably represents the decompensation (instability) of the critical condition, leading to a transition to the next phase of chronicity. It should be noted that in the epidemiological study by Iwashyna TJ et al. (2016) involving over 1,000,000 patients, there was a demonstrated progressive decline in the prognostic power of the etiological factor of acute pathology in predicting mortality as the length of hospitalization increased [73]. On average, by day 10, when the second peak of ICIS incidence occurs in our study, the characteristics of the etiological factor of acute pathology in prognostic terms become indistinguishable from standard anamnestic data of patients, such as sex, age, and overall comorbidity [73]. On an individual level, this likely manifests in the development of the third step of the chronic critical state, where the severity of the condition is no longer dictated by the acute pathological process but rather by the progression of typical pathological processes.

Addressing the strengths and weaknesses of this study, its major strength lies in being conducted using an open database containing data on more than 100,000 patients. However, a limitation is the retrospective design of the study and the insufficient amount of clinical data that could enhance the understanding of the chronicity process of critical conditions. Additionally, the current absence of a unified definition of inflammation, immunosuppression, and catabolism syndrome may reduce the internal validity of the study when a unified definition is established in the future. Despite the high external validity of our study results using routinely assessed ICU markers, the lack of a standardized definition underscores the need for large-scale prospective observational studies to identify the most sensitive biomarkers of inflammation, immunosuppression, and catabolism.

Our findings reinforce the notion that ICIS acts as a trigger for extended and chronic critical illness. This hypothesis is further supported by the recent systematic review conducted by Chadda et al. [74]. Based on these findings, further research into chronic critical illness and the ICIS triad is warranted.

## 5. Conclusions

The development of the ICIS triad during hospitalization increases the risk of mortality by 2.5 times (*p* = 0.009) and reflects the chronicity of critical conditions. The occurrence of two peaks in the development of this triad provides grounds for introducing a new Tri-steps model of critical condition chronicity, encompassing acute, extended, and chronic phases.

## Figures and Tables

**Table 1:** Initial parameters, disease characteristics, and outcomes.

Parameters		ICIS, N = 60	No ICIS, N = 834	*p* Value
Sex	Male	37, 57%	448, 54%	0.7 ^1^
	Female	26, 43%	386, 46%	
Age, years		63.5 (IQR 56.3–74.0)	63.5 (IQR 52.0–73.0)	0.5 ^2^
BMI, kg/m^2^		26.2 (IQR 21.0–32.7)	27.5 (IQR 23.2–33.5)	0.18 ^2^
Body weight on admission, kg		79 (IQR 62–99)	81 (IQR 67–97)	0.4 ^2^
Body weight at discharge, kg		78 (IQR 48–98)	79 (IQR 65–96)	0.3 ^2^
Change in body weight, kg		0.4 (IQR −50.2–6.3)	0.0 (IQR −4.1–3.2)	0.8 ^2^
APACHE IV, score		71 (IQR 46–91)	59 (IQR 45–76)	0.017 ^2^
Mechanical ventilation (MV)		27, 45%	273, 33%	0.052 ^1^
Duration of MV, days		5 (IQR 2–13)	4 (IQR 2–10)	0.5 ^2^
Use of vasoactive drugs		16, 27%	117, 14%	0.008 ^1^
Lactate level on admission, mmol/L		1.8 (IQR 1.3–3.0)	1.5 (IQR 1.0–2.4)	0.068 ^2^
Main reasons for ICU admission				
Sepsis		14, 23%	250, 30%	0.3 ^1^
Cardiac arrest		1, 1.7%	20, 2.4%	0.9 ^3^
Respiratory arrest		0, 0%	15, 1.8%	0.6 ^3^
Coma		0, 0%	17, 2.0%	0.6 ^3^
Bleeding		2, 3.3%	20, 2.4%	0.7 ^3^
Cardiogenic shock		0, 0%	4, 0.5%	0.9 ^3^
Hypovolemia		0, 0%	8, 1.0%	0.9 ^3^
Pancreatitis		4, 6.7%	12, 1.4%	0.018 ^3^
Acute renal failure		2, 3.3%	21, 2.5%	0.7 ^3^
Congestive heart failure		2, 3.3%	34, 4.1%	0.9 ^3^
Pneumonia		5, 8.3%	30, 3.6%	0.079 ^3^
Weaning from MV		1, 1.7%	28, 3.4%	0.7 ^3^
Acute myocardial infarction		0, 0%	17, 2.0%	0.6 ^3^
Seizures		0, 0%	12, 1.4%	0.9 ^3^
Supraventricular arrhythmias		1, 1.7%	17, 2.0%	0.9 ^3^
Stroke		0, 0%	15, 1.8%	0.6 ^3^
Cardiac surgery		1, 1.7%	13, 1.6%	0.9 ^3^
Hospitalization outcomes				
Hospital mortality		11, 18.3%	41, 4.9%	<0.001 ^3^
ICU mortality		11, 18.3%	41, 4.9%	<0.001 ^3^
Duration of hospitalization, days		13 (IQR 9–19)	11 (IQR 6–18)	0.041 ^2^
Length of ICU stay, days		6.0 (IQR 2.7–13.4)	3.4 (IQR 1.8–8.3)	<0.001 ^2^
Discharge location	Home	17, 45%	357, 50%	0.5 ^1^
	Other (rehabilitation, other hospital)	21, 55%	354, 50%	

^1^—Chi-square test; ^2^—Mann-Whitney U test; ^3^—Fisher’s exact test.

**Table 2:** Multivariable Cox regression analysis—predictors of the ICIS.

Parameters	AUROC (95% CI)	Hazard Ratio (95% CI)		*p* Value
APACHE IV	0.598 (0.512–0.684)	APACHE IV ≥ 65 *	2.36 (1.33–4.18)	0.003
		No cutoff point	1.013 (1.003–1.022)	0.012
Pancreatitis	-	3.07 (0.94–10.1) *		0.063

* Included in the final model.

## Data Availability

Restrictions apply to the availability of these data. Data were obtained from eICU-CRD and are available at https://physionet.org/content/eicu-crd/2.0/ (accessed on 1 May 2024) with the permission of PhysioNet.

## Supplementary Materials

The following supporting information can be downloaded at: https://www.mdpi.com/article/10.3390/jcm13133683/s1, File S1. STROBE checklist.
